# ASSOCIATIONS BETWEEN CLINICAL FUNCTIONING AND AD BIOMARKERS AMONG HISPANIC AND WHITE NON-HISPANIC OLDER ADULTS

**DOI:** 10.1093/geroni/igad104.0568

**Published:** 2023-12-21

**Authors:** Miriam Rodriguez, Lisandra Mendoza, Patricia Garcia, Andres Duarte, Dilianna Padron, Michael Marsiske, Jacob Fiala, Ranjan Duara

**Affiliations:** Indiana University Bloomington, Bloomington, Indiana, United States; Bay Pines VA Health System, Bay Pines, Florida, United States; Indiana University School of Medicine, Indianapolis, Indiana, United States; Albizu University, Doral, Florida, United States; Central Virginia VA Healthcare System, Richmond, Virginia, United States; University of Florida, Gainesville, Florida, United States; University of Florida, Gainesville, Florida, United States; Mount Sinai Medical Center, Miami Beach, Miami Beach, Florida, United States

## Abstract

**Objectives:**

Hispanics are 1.5x more likely to develop Alzheimer’s disease (AD) when compared to White non-Hispanics (WNHs). There is also evidence to support that cognitive performance disproportionately reflects neuropathology among Hispanics and that functional decline is concurrent with the accumulation of AD biomarkers. The current study aimed to examine relationships between AD biomarkers and a functional measure among Hispanic and WNH older adults. It was hypothesized that the functional measure would be strongly related to AD biomarkers among Hispanics.

**Methods:**

The modified clinical dementia rating scale (mCDR) was administered in the participants primary language (English or Spanish) to WNH (n=203) and Hispanic (n=258) older adults who were cognitive normal or diagnosed with Mild Cognitive Impairment (MCI) or dementia. Invariance SEM models were used to compare the pattern of relationships between the mCDR and neurocognitive test performance, MRI volumes, and amyloid load adjusting for age, education, ApoE4 status, and intracranial volume.

**Results:**

Model fit was good and not significantly worsened by imposing strict structural invariance. Nested model comparisons indicated that regression weights and correlations among measures differed by group, suggestive of moderation by Hispanic status. Among Hispanic participants, sex (♌=-0.17, p<.05) and Amyloid load (♌=0.25, p<.001) significantly predicted mCDR scores. MRI volumes significantly predicted MCDR scores among both Hispanic (♌=-0.51, p<.001) and WNH participants (♌= -0.42, p<.001).

**Conclusions:**

Functional measures like the mCDR may better correlate with Amyloid load among Hispanic older adults than among WNHs, while the correlation with MRI volumes may be comparable in both groups.

